# Mapping future fire probability under climate change: Does vegetation matter?

**DOI:** 10.1371/journal.pone.0201680

**Published:** 2018-08-06

**Authors:** Alexandra D. Syphard, Timothy Sheehan, Heather Rustigian-Romsos, Kenneth Ferschweiler

**Affiliations:** Conservation Biology Institute, Corvallis, Oregon, United States of America; Centro de Investigacion Cientifica y de Educacion Superior de Ensenada Division de Fisica Aplicada, MEXICO

## Abstract

Understanding where and how fire patterns may change is critical for management and policy decision-making. To map future fire patterns, statistical correlative models are typically developed, which associate observed fire locations with recent climate maps, and are then applied to maps of future climate projections. A potential source of uncertainty is the common omission of static or dynamic vegetation as predictor variables. We therefore assessed the sensitivity of future fire projections to different combinations of vegetation maps used as explanatory variables in a statistically based fire modeling framework. We compared models without vegetation to models that incorporated static vegetation maps and that included output from a dynamic vegetation model that imposed three scenarios of fire and one scenario of land use change. We mapped projected future probability of all and large fires (> = 40 ha) under two climate scenarios in a heterogeneous study area spanning a large elevational gradient in the Sierra Nevada, California, USA. Results showed high model sensitivity to the treatment of vegetation as a predictor variable, particularly for models of large fire probability and for models accounting for wildfire effects on vegetation, which lowered future fire probability. Some scenarios resulted in opposite directional trends in the extent and probability of future fire, which could have serious implications for policy and management resource allocation. Model sensitivity resulted from high relative importance of vegetation variables in the baseline models and from large predicted changes in vegetation, particularly when simulating wildfire. Although statistical fire models often omit vegetation due to uncertainty, model sensitivity demonstrated here suggests a need to account for that uncertainty. Coupling statistical and processed based models may be a promising approach to reflect a more plausible range of scenarios.

## Introduction

The scientific community is reaching growing consensus that global climate change will significantly alter future wildfire dynamics, e.g., [1–3]. Thus, a key research and management challenge is to project and map potential future changes in the extent and location of wildfire patterns. Understanding where and how fire patterns may change in the future is critical for fire managers and policy makers trying to develop strategies and anticipate resources needed to prepare for these changes.

Fire patterns emerge over space and time according to regionally distinctive fire regimes, e.g., via variation in fire size, frequency, area burned, and severity; which derive from a landscape’s climatic, topographic, and vegetative characteristics, plus the timing and distribution of lightning or human-caused ignitions [4–6]. Therefore, the idea behind mapping the spatial manifestation of future fire regimes is to model the relationships among and mechanisms behind the primary drivers of recent fire activity, particularly climate, to predict future fire activity as a function of changes in the drivers.

The two primary methodological approaches used for mapping future fire activity include statistical-correlative models and process-based simulation models, although projections based on changes in fire danger indices have also been developed [7–9]. The statistical approach is similar to species distribution modeling [10] in that response functions are estimated based on observations of fire occurrence or frequency in relation to their hypothesized climatic or other human or biophysical environmental drivers [11–12]. Fitted models conditioned upon point locations reflecting recent conditions are then used to map the likelihood of fire at unmapped locations in space and time; future fire patterns are mapped by substituting projected future climate maps for the current climate variables used to fit the model [13–14].

Advantages of this top-down statistical approach are that these models are relatively simple to parameterize and provide intuitive mapped output; they provide an assessment of variable importance, thus contributing to theoretical understanding of fire-climate relationships; and they efficiently enable comparisons of alternative climate or management scenarios. Major drawbacks include the assumption of stationary relationships over time and the inability to account for dynamic feedbacks among fire, climate, vegetation, and human activity [9,15–16]. In particular, the common practice of omitting vegetation dynamics could introduce a major source of uncertainty into model projections, as fuel moisture, abundance, and structure are key controls over fire activity [17]. Climate change is expected to independently result in dramatic, yet uncertain plant species range shifts due to physiological constraints [18,19]. Fire also feeds back strongly with climate and vegetation patterns [1,16,20].

Despite the importance of vegetation in controlling fire, the overwhelming amount of uncertainty in future vegetation dynamics [21] is one argument for excluding vegetation in future fire mapping efforts [22]. On the other hand, some efforts have accounted for the spatial distribution of broad fuel types or characteristics by using a static map of vegetation or net primary productivity as a predictor variable in the statistical models and keeping the map constant while climate variables change [9,14,23]. An alternative approach for explicitly accounting for vegetation has been to assume that certain climate covariates can implicitly account for the primary climatic controls over vegetation that are relevant to fire behavior [9,24]. Fire activity tends to be highest at intermediate levels of productivity, resulting from distinctive combinations of temperature and moisture [25–27]. These combinations can be quantified via indices of climatic water balance (i.e., via actual evapotranspiration (AET)) and fuel moisture (i.e., climatic water deficit (CWD)) and used to assume future vegetation conditions [28–29].

The primary alternative to statistical mapping of future fire activity is to use process-based dynamic global vegetation models (DGVMs) that can explicitly represent and simulate fire-climate-vegetation interactions and feedbacks. These models can simulate the complex nonlinearities and feedbacks that are not possible with static correlational models, however, the geographical distribution of fire in these models remains relatively crude, in part due to the typically coarse resolution associated with modeling on a regional to global scale [25,30–31].

In addition to climate, vegetation, and topography (which is not difficult to account for, as it remains static over time), land use and land cover change may also be significant drivers of future fire activity. Land use patterns, particularly urban development, are spatially correlated with human ignitions [32–34], and land cover change imposes additional modifications to fuel conditions and continuity [35–37]. Human influence, in addition to climate, has been shown to be a significant factor in controlling fire patterns in a statistical modeling approach applied to North America [38]. While few statistical projections of future fire patterns account for dynamic land use change, one study incorporated forecasts of housing extent and density with climate change projections, and results showed that up to 50% of the variation in future fire patterns was due to anthropogenic factors [29].

Given that sound management and policy decisions require plausible maps of fire activity patterns [5], it is important to understand the range of uncertainty imposed by excluding vegetation from climate-based fire projection maps. Therefore, our objective was to assess differences in future fire projections as a result of using different combinations of vegetation maps, in addition to climate and topography, as explanatory variables in a statistically based fire modeling framework. We compared models without vegetation with models that incorporated static vegetation maps in addition to models that included dynamic output vegetation maps from a DGVM that imposed three scenarios of fire (no fire, fire suppression, and fire without suppression). We also ran the DGVM with and without projections of land use change. We mapped the projected future probability of all fires (of any size) and large fires (> = 40 ha) under two climate scenarios in a heterogeneous study area spanning a large elevational gradient in the Sierra Nevada, California, USA.

We asked:

1) How important is vegetation relative to climate, topography, and land use in statistical-correlative models of fire distribution?
2) Does the explicit integration of vegetation lead to different conclusions about future fire patterns?
3) Are there differences in projections based on the use of static versus dynamic vegetation inputs or dynamic simulations of fire?
4) Are any projected differences in projections mediated by climate scenario or size of fire modeled?

## Materials and methods

### Study area

**Fig 1:**
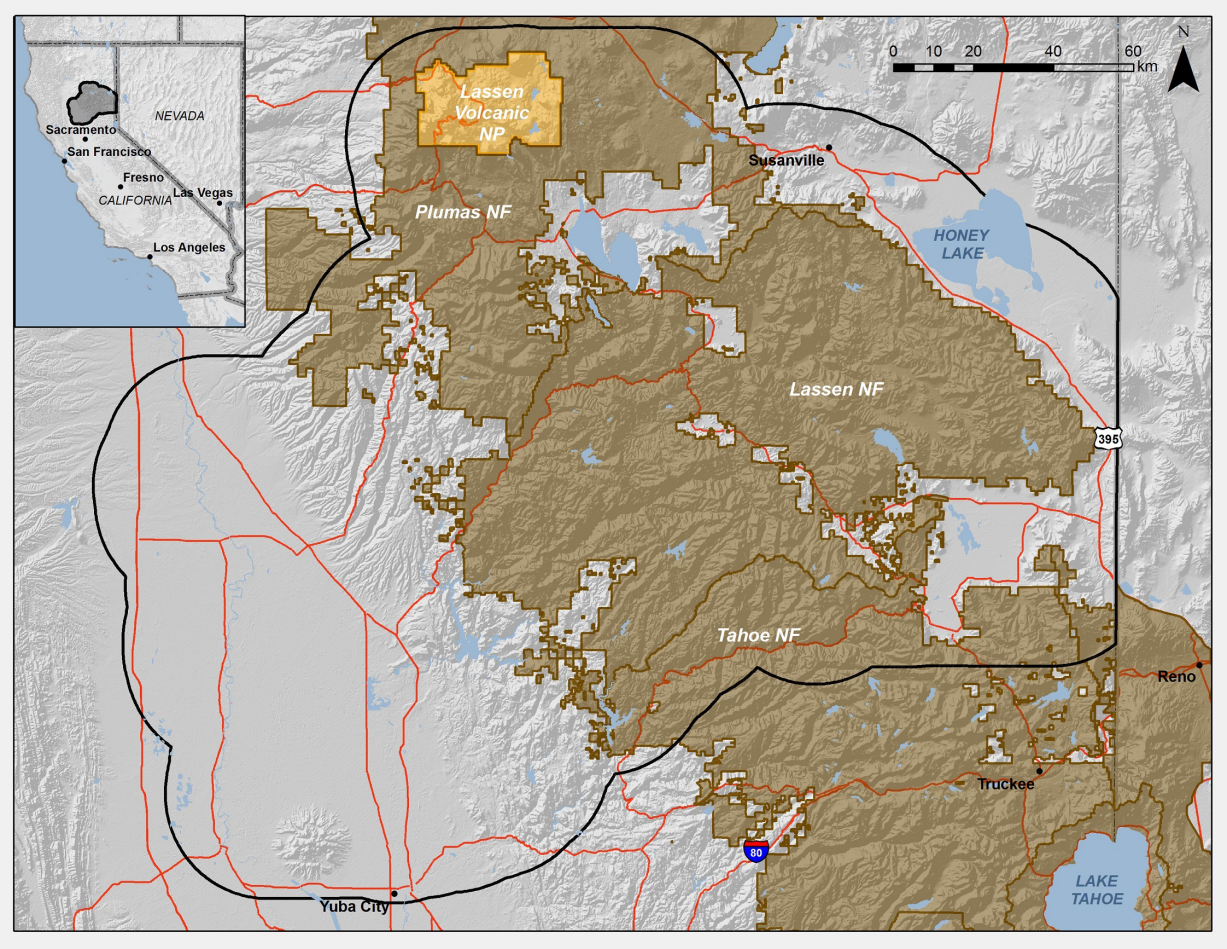
a) Butte and Plumas Counties study area in the Sierra Nevada, California, USA, and b) elevation (meters). Sources of base map: National Geographic, Esri, DeLorme, HERE, UNEP-WCMC, USGS, NASA, ESA, METI, NRCAN, GEBCO, NOAA, iPC.

The study area included Butte and Plumas Counties, in addition to a 20 km buffer surrounding them (2,277,679 ha of land), on the western slope of the northern Sierra Nevada, California where the range transitions to the southern end of the Cascades (Fig 1A). The region is biophysically heterogeneous and spans an elevational gradient from 11m in the Central Valley to 3128m in Lassen Volcanic National Park (Fig 1B). The climate is primarily Mediterranean with most of the precipitation occurring in winter, and there is a strong climatic gradient from west to east. Vegetation is also diverse, with grassland, shrubland, and mixed forest dominating the lower-elevation foothills where most of the development is located; conifer forest interspersed with mixed forest and shrublands dominating in the highest elevations; and shrublands again covering most of the lower-elevation eastern slopes of the mountains. Much of the landscape is protected in the Lassen, Plumas, and Tahoe National Forests and Lassen Volcanic National Park, but there has also been substantial residential development in the foothills, extending out of the Chico, CA metropolitan area. Wildfire is an important and frequent natural ecological process in the region. Human-caused ignitions dominate in the lower elevations while lightning is more common in the higher-elevation forests. Recent wildfires in 2008, 2015, and 2017 resulted in more than 1000 structures destroyed and two lost lives in Butte County.

### Data

We modeled projections of future fire distributions using a range of topographic, climatic, anthropogenic, and vegetation explanatory variables (Table 1). After considering a larger range of potential variables than those listed below, we used ENMTools [39] to calculate Pearson correlation coefficient between predictors for each baseline model (described below). For predictors with values r > = 0.8, we selected the variable with higher mean 5-fold cross validated area under the receiver operating characteristic curve (AUC), a threshold-independent assessment of model discriminatory ability [40] in a univariate model. While elevation did perform better than highly correlated climate variables for large fires, we opted to drop it to maximize sensitivity to climate projections. To match the resolution of the climate data, we resampled all of the gridded explanatory variables to 270m.

**Table 1:** Description of variables used for statistical modeling of fire patterns in Butte and Plumas Counties, CA.

*Variable*	Description and source	Range and units
*Fire (Dependent variable)*		
*All fires*	Fire occurrence locations 2000–2010 (Short 2014)	0.004–26302.321 ha, mean 23.430
*Large fires*	Cal Fire fire perimeter database	41.624–26288.361 ha, mean 1611.678
*Terrain*		
*Slope*	LANDFIRE, 30-m native resolution, aggregated by mean to 270m	0–48.23; %
*Solar insolation index*	Derived from LANDFIRE elevation data: s = 2 –(sin((slope/90)180))*(cos(22 –aspect) + 1), aggregated by mean to 270m	0.07–2.00, unitless index
*Southwestness*	Derived from LANDFIRE elevation data: s = cos (aspect_lf—255), aggregated by mean to 270m	-0.58–0.49; unitless index
*Climate (1980–2010 normals)*		
*Temperature seasonality*	Coefficient of variation across calendar year of temperatures	0.021–0.028; Kelvin
*Annual minimum temperature*	Mean low temperature of coldest month	-12.14–4.54; °C
*Annual precipitation*	Sum over calendar year	131.43–2903.86; mm
*Summer precipitation*	Sum over June, July, August	4.64–57.15; mm
*Climatic Water Deficit*	Potential minus actual evapotranspiration	0–1021.77; mm
*Vegetation*		
*Vegetation type*	Landfire existing vegetation	
*Vegetation type*	Output from DGVM	
*Dead wood*	Output from DGVM	0–12551.9; g C m-2
*Standing dead grass carbon*	Output from DGVM	0–120.963; g C m-2
*Forest carbon*	Output from DGVM	0.002–34261.4; g C m-2
*Land use effects*	Sleeter et al., 2017	Classes
*Anthropogenic*		
*Distance to roads*	TIGER line, Exclude 4WD and OHV roads; Combine others, including RRs. TIGER Roads 2015, U.S. Department of Commerce, U.S. Census Bureau, https://www.census.gov/geo/maps-data/data/tiger.html	0–7521.3; m
*Distance to development*	Extract developed areas from land use maps, calculate distance to development for current and each predicted time step (Sleeter et al., 2017)	0–46131.3; m

#### Fire data

Given that conditions needed for fires to become large may be different than conditions for fire to simply occur [11,41–42], we developed and ran statistical projections based on two sources of fire data (Table 1). We compiled the first dataset, hereafter “all fires,” from the National Interagency Fire Program Analysis, Fire-Occurrence Database (FPA FOD) [43], which includes the spatial coordinate information indicating the point of ignition for all fires across all land ownership types, with the date and size of fire included as attributes. We overlaid and selected all fires that occurred within the study area for the years 2000–2010, to ensure we developed models based on recent environmental controls. To minimize the potential influence of spatial autocorrelation, we compared four minimum nearest neighbor distance filter sizes for thinning the points (500m and 1, 2, and 5 km). The 500m performed slightly better than the others (best balanced sensitivity and area predicted suitable), reducing the sample size from 7238 to 2048.

For the dataset of large fires, we used the State of California Fire and Resource Assessment Program (FRAP) fire history database that provides spatially explicit wildfire perimeter data for all moderate-large fires reported by state and federal agencies. Again using data from 2000–2010 and overlaying the perimeters with the study area, we generated a random sample of points within perimeters of fires > = 40 ha according to the method developed by Davis et al. [22]. That is, within each wildfire, we generated the number of points per fire proportional to the square root of the ratio between perimeter area and area of smallest fire, which resulted in a sample size of 346. We also forced a minimum distance of 270m between the random points generated so there would be no ‘duplicate’ points within a single grid cell.

#### Topographic data

We evaluated three topographic variables to account for the effect of terrain on soil moisture and development, energy balance, and in turn, fuel characteristics and flammability [10,44–45] (Table 1). To account for the role of terrain in moderating energy and moisture balance, and in distinguishing xeric from mesic exposures, we used an index of solar insolation [46] as well as ‘southwestness’ [45], which is a cosine transformation of aspect. We also included slope, as this variable is also important in moderating fire behavior and spread rates [17,44]. The slope and baseline elevation and aspect variables were acquired from LANDFIRE [47].

#### Climate data

We evaluated a range of climatic variables representing energy and moisture gradients that have been significantly associated with fire patterns in other studies e.g., [9,13,22,38, 48–49] (Table 1). These included two temperature variables, annual minimum temperature and temperature seasonality; two moisture variables, annual and summer precipitation; and climatic water deficit (CWD), which is an integrative measure of climatic and edaphic factors that are indicative of drought stress [50–51].

The climate data were available as annual historical and projected surface grids developed by the California Basin Characterization Model (CA-BCM 2014) at a 270-m resolution (https://cida.usgs.gov/thredds/CA-BCM-Catalog.html) [51]. Historical PRISM temperature and precipitation data [52] were downscaled from 800-m resolution using Gradient-Inverse-Distance-Squared (GIDS) downscaling [53]. We processed these annual data to create 30-year baseline statistical summaries from 1980–2010. We compared future climate projections using climate surfaces from two CMIP-5 General Circulation Models that have been recommended as priorities for research due to their relevance to California and their range of possible futures [54]. These included CNRM-CM5 (“cool/wet”) and MIROC5 (complement/cover range of outputs). For both scenarios, we used the RCP 8.5 “business as usual” emissions scenario. We processed the data for projections at three time steps (2010–2039, 2040–2069, and 2070–2099, hereafter 2010, 2040, and 2070, respectively).

#### Vegetation data and output from MC2 DGVM

To generate dynamic projections of vegetation to use as input to the statistical model, we ran the MC2 dynamic global vegetation model (DGVM) to create decadal mapped outputs of vegetation type and biomass. MC2 [55] is a process-based model used at global to regional scales to simulate potential vegetation, carbon fluxes and pools, and wildfire. MC2 runs on a monthly timestep over a spatial grid, with each grid cell simulated independently.

Three modules simulate biogeochemistry, biogeography, and wildfire. The biogeochemistry module simulates water, carbon, and nutrient flows between pools based on climate, soil, and starting state. The biogeography component of the model determines the potential life forms and dynamic vegetation types on the landscape, which are defined based on a set of climate and biomass threshold rules. In the model simulations, vegetation types (not species) shift in response to climate change and atmospheric CO_2_ concentration. Competition between woody vegetation and grass is simulated as a function of light, nitrogen, and available soil water. The model generates output for various live and dead carbon pools, carbon fluxes, fuel characteristics, hydrologic fluxes, and vegetation characteristics.

Fire is simulated as a discrete event with impacts including mortality and aboveground biomass consumption. A wildfire is triggered when a cell’s fuel condition exceeds its vegetation type’s fuel threshold. Ignitions are assumed and a cell is limited to one fire per year. Fire suppression effectively sets a vegetation type’s fuel condition threshold higher under the assumption that fires occurring under less severe fuel conditions can be extinguished. The fraction of a cell burned is limited to the years since last fire divided by its vegetation type’s fire return interval.

Land use land cover (LULC) inputs used with the MC2 simulations were Sleeter et al.’s [56]; hereafter Sleeter) business as usual (BAU) scenario projections. We aggregated Sleeter’s development and transportation development classes into a single developed class and aggregated all classes unaffected by human activity into natural. This produced five land use classes for the model: developed, mining, annual agriculture, perennial agriculture, and natural. Within MC2, when land is converted from natural to one of the other classes, all above- and belowground vegetation components are removed. No vegetation grows in developed and mining cells. Annual crops grow as grasses and harvest removes all aboveground live carbon, 90% of aboveground dead carbon, 50% of belowground dead carbon. 95% of belowground living carbon is killed. Perennial crops grow as wild, with no harvest their first five years, removal of 90% dead carbon and 50% of live growing season production during years 5–40, and complete removal and restart after year 40. Fire is excluded from croplands.

MC2 is run in four separate phases: *equilibrium* to attain stable carbon pools and vegetation cover; *spinup* to readjust carbon pools and vegetation types in response to dynamic fire; *historical transient*, driven by observation based climate data; and *future transient*, driven by projected future climate data. For this study, we ran MC2 across the entire state of California at a 30 arc second (approximately 800 m) resolution using observation-based historical climate data from 1895 to 2010, and and future climate projections from 2011 to 2099. Results were clipped the output to the extent of our study area. Outputs were resampled to match the climate data grid. To account for a range of potential fire and land use effects on vegetation and biomass, we ran the model under combinations of three different fire scenarios (no fire, fire suppression, and full fire) and with and without land use change incorporated into the simulations (Table 2). The fire and land use scenarios were applied over the entire duration of the MC2 simulations, from 1895 through 2100. Thus, vegetation change could be observable before the simulation of future climate and land use projections.

**Table 2:** Name and description of vegetation scenarios used for statistical fire mapping projections. All eight scenarios were also modeled under two climate change scenarios (MIROC and CNRM) and for two sources of fire data (all fires and large fires).

*Scenario name*	*Abbreviation*	*Baseline for projections*	*Vegetation*	*Time variant*	*Fire*	*Land use*
*No vegetation*	NoVeg	NoVeg	None	—	—	—
*Mapped static*	MapStat	MapStat	Mapped veg	Static	—	—
*Modeled static*	ModStat	ModStat	Modeled veg	Static	—	—
*Modeled dynamic*	ModDyn	ModStat	Modeled veg	Dynamic	—	—
*Modeled dynamic land use*	ModDynLU	ModStat	Modeled veg	Dynamic	—	Land use
*Modeled dynamic no fire*	ModDynNF	ModStat	Modeled veg	Dynamic	—	—
*Modeled dynamic no fire land use*	ModDynNF	ModStat	Modeled veg	Dynamic	—	Land use
*Modeled dynamic full fire*	ModDynFire	ModStat	Modeled veg	Dynamic	Fire	
*Modeled dynamic suppressed fire*	ModDynFS	ModStat	Modeled veg	Dynamic	Suppressed	—
*Modeled dynamic suppressed fire land use*	ModDynFSLU	ModStat	Modeled veg	Dynamic	Suppressed	Land use

Our objective was to compare results among scenarios with three different assumptions regarding vegetation: no explicit vegetation (NoVeg), static vegetation (MapStat and ModStat (MC2)), and simulated dynamic vegetation (ModDyn (MC2); Table 2). We first evaluated a map of existing vegetation type (LANDFIRE [57]), (MapStat, Table 2), then cross-walked reclassified vegetation types from that map to classes produced as output from MC2 to attain the best comparability in simulated current conditions (S1 Table). To attain maximum map agreement and simplicity for the statistical modeling, we consolidated vegetation types into four broad vegetation types: grassland, shrubland, mixed and broadleaf forest, and needleleaf forest. We merged and labeled the rest of the classes as nonflammable, including development, agriculture, water, and barren land.

Because MC2 produces continuous model output that may be more directly related to potential fire behavior than classified vegetation types, we also evaluated several of these continuous variables as potential predictors in the statistical models: Dead wood carbon; total ecosystem carbon; live aboveground carbon; and standing dead grass carbon.

#### Anthropogenic data

Given that human infrastructure, including development and roads, are spatially associated with ignition and fuel continuity patterns [11,42,58], we used two anthropogenic explanatory variables in all scenarios: Euclidean distance to roads and dynamic distance to urban development. To develop the distance to urban development data, we extracted the “development” land use type from the Sleeter land use projections and calculated Euclidean surfaces to this binary land use type. We used 2010 development data for baseline models and the 2010–2039 period, 2040 development data for the 2040–2069 period, and 2070 development data for 2070–2099 period.

### Statistical modeling

To project future fire patterns under the alternative vegetation scenarios, we used the statistical machine learning method, MaxEnt [59–60]. The model algorithm generates 10,000 randomly located background points and then iteratively evaluates contrasts between values of the environmental predictor variables at these background locations with values of the predictors at the locations of the dependent variable (in this case, all or large fires). The best approximation of the distribution is determined as the one with maximum entropy. The output is an exponential function that assigns a probability of occurrence to each site or cell of a map. MaxEnt is widely used in many fields, with thousands of applications published in scientific journals, including for mapping the distribution of all or large fires [22,24,38,48]. Its wide use is largely due to its reputation as one of the top-performing modeling methods for probabilistic distribution modeling [61–63].

For all scenarios, we used MaxEnt version 3.3.3k with the default settings, except we excluded product and threshold features, did not use clamping, and used a regularization multiplier of 1.5 to minimize overfitting. We developed three different baseline models for both all fires and large fires to correspond with the three versions of vegetation used to establish historical conditions. These included a baseline with no vegetation, a baseline using the LANDFIRE existing vegetation type map, and a baseline using the 2010 vegetation output of the MC2 model. We ran the baselines with 5 cross-validated replicates to get a mean 5-fold CV AUC and permutation importance values. For the future projections under different climate and vegetation scenarios, we projected the baseline models onto maps of future conditions for all time steps.

#### Analysis

To compare all scenarios, we first calculated the total predicted suitable area of all and large fires by applying a threshold to create binary maps. We used a threshold of maximum sensitivity plus specificity because it has been shown to optimize discrimination between presence and absence data [64]. From the resulting binary maps for all scenarios and time steps, we then summed the total area predicted suitable. In addition to the area predicted suitable for fires, we calculated the mean probability of all or large fires for all scenarios by summing the total predicted probabilities across all grid cells then dividing by the number of cells in the map.

To characterize the detailed effects of modeled vegetation and related processes on large fire probability, we compared NoVeg fire probabilities with those from three scenarios with vegetation (WithVeg) ModStatFireLU, ModDynFireLU, and ModDynLU for each of the three time periods and each climate future. Difference maps were produced by subtracting NoVeg probabilities from each of the WithVeg scenarios. Differences were tabulated for each combination of: vegetation type, time period, scenario, climate future. Aggregate distributions of probability differences were plotted by vegetation type for each climate future.

## Results

### Climate and vegetation modeling results

Over the entire region, compared to historical values, CNRM projected increases in annual and summer precipitation throughout the 21^st^ c. with a maximum annual increase of 42% at the end of the century (S2 Table). MIROC, on the other hand, projected slightly higher annual precipitation initially followed by lower precipitation, with a decrease of 17% by the end of the century, although summer precipitation was projected to increase. These patterns were generally consistent across elevations. Minimum temperatures were projected to rise by several degrees for both scenarios over the 21^st^ c. for all elevations, with end of century CNRM rises being 0.4, 0.16, and 0.76 greater than MIROC’s for the full study area, elevations less than or equal to 1000 m, and elevations greater than 1000 m, respectively. Climatic water deficit (CWD) was also projected to increase by the end of the century for both scenarios and across elevations, with CNRM rising less compared to MIROC (11% and 24% for CNRM and MIROC, respectively, over the whole area). CWD initially declined for CNRM at all elevations before increasing.

After reclassification of broad vegetation types, there was 73% agreement between MC2 baseline and LANDFIRE vegetation maps. Compared with LANDFIRE, MC2 vegetation had more shrubland in the Central Valley and Cascade foothills, with less nonflammable area in the Central Valley and less mixed and broadleaf cover at lower elevation. MC2 baseline had more mixed and broadleaf ranging higher into the Cascade foothills where needleaf dominated under LANDFIRE. Shrubs were more present in the northwestern portion of the study region under MC2 versus LANDFIRE. MC2 delineated grasslands only in areas recovering from simulated timber harvest while LANDFIRE mapped grassland along the edges of the Central Valley.

**Fig 2:**
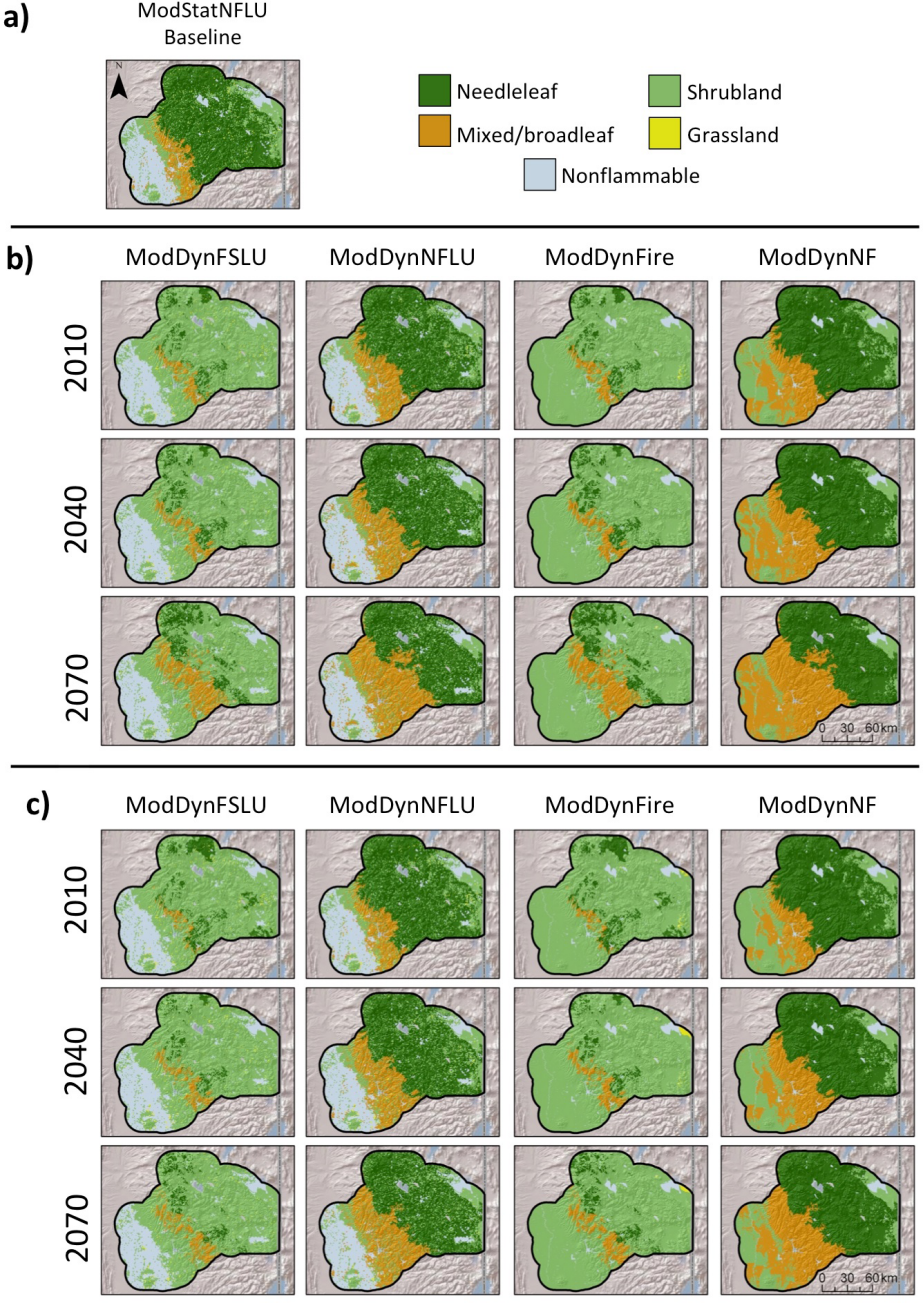
Baseline and projected future vegetation type from MC2 runs for selected scenarios with a) Baseline and b) CNRM and c) MIROC climate futures.

For scenarios with vegetation change, there were large differences between those with fire and those without, with substantial differences already apparent by 2010, after running the simulations with different treatments from 1895 (Fig 2). For example, under the with-fire scenarios (ModDynFSLU and ModDynFire), the study region was dominated by shrubland to the near exclusion of all other classes, except nonflammable for ModDynFSLU. Under the no-fire scenarios (ModDyneNFLU and ModDynNF), the region was dominated by a combination of needleleaf and mixed and broadleaf forests. Generally, CNRM projected more mixed and broadleaf forest than MIROC, which had greater needleaf forest in the no-fire scenarios and greater shrub in the with-fire scenarios.

**Fig 3:**
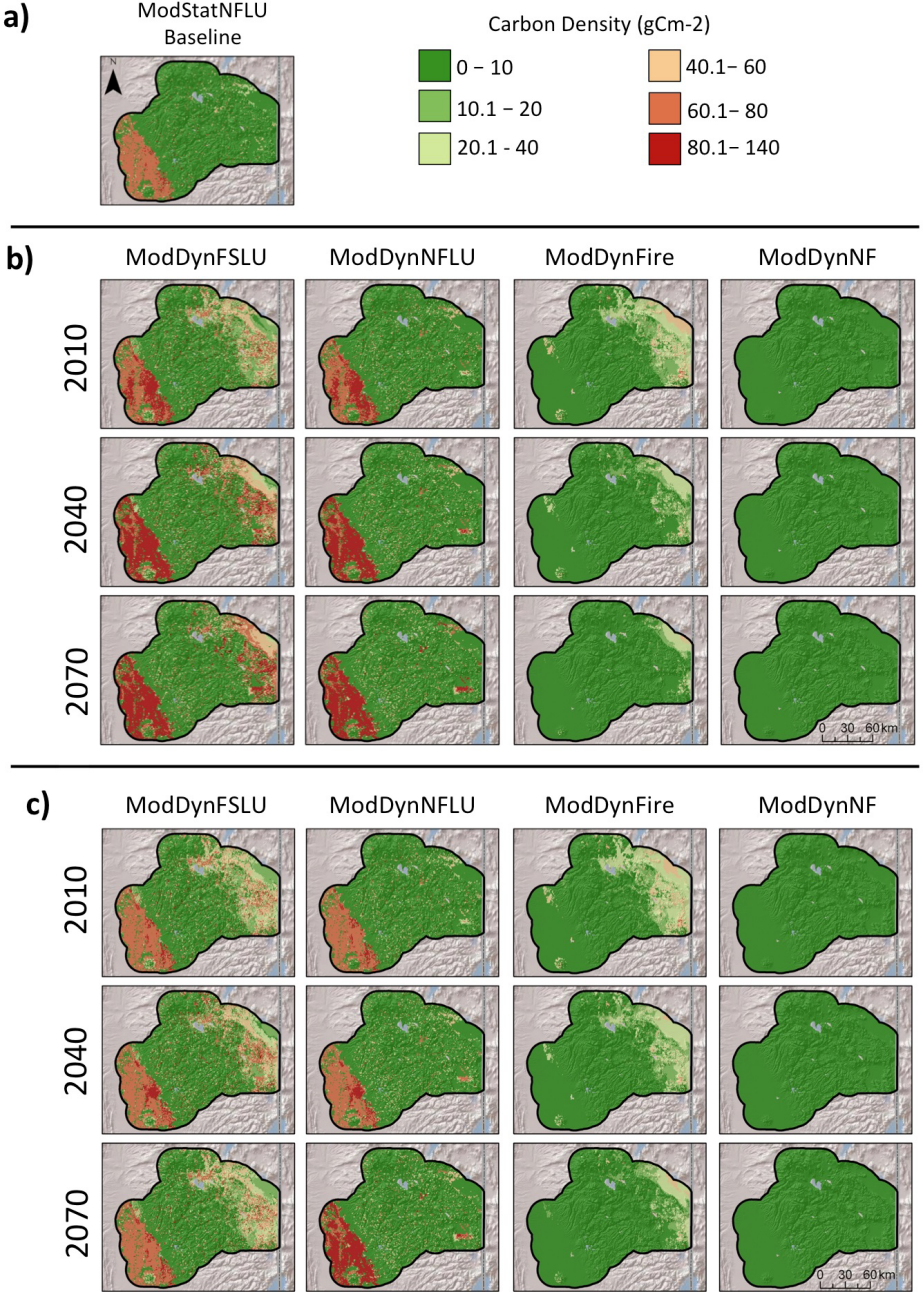
Maximum annual carbon in standing dead grass from MC2 runs for selected scenarios with a) Baseline and b) CNRM, and c) MIROC climate futures.

Maximum annual carbon in standing dead grass (Fig 3) was highest in the Central Valley portion of the study area in the land use change scenarios (ModDynFSLU and ModDynNFLU), and this was greater under CNRM than under MIROC. It was also somewhat higher in the western portion of the study in the with-fire scenarios, with larger areal extent under MIROC but higher values with CNRM.

**Fig 4:**
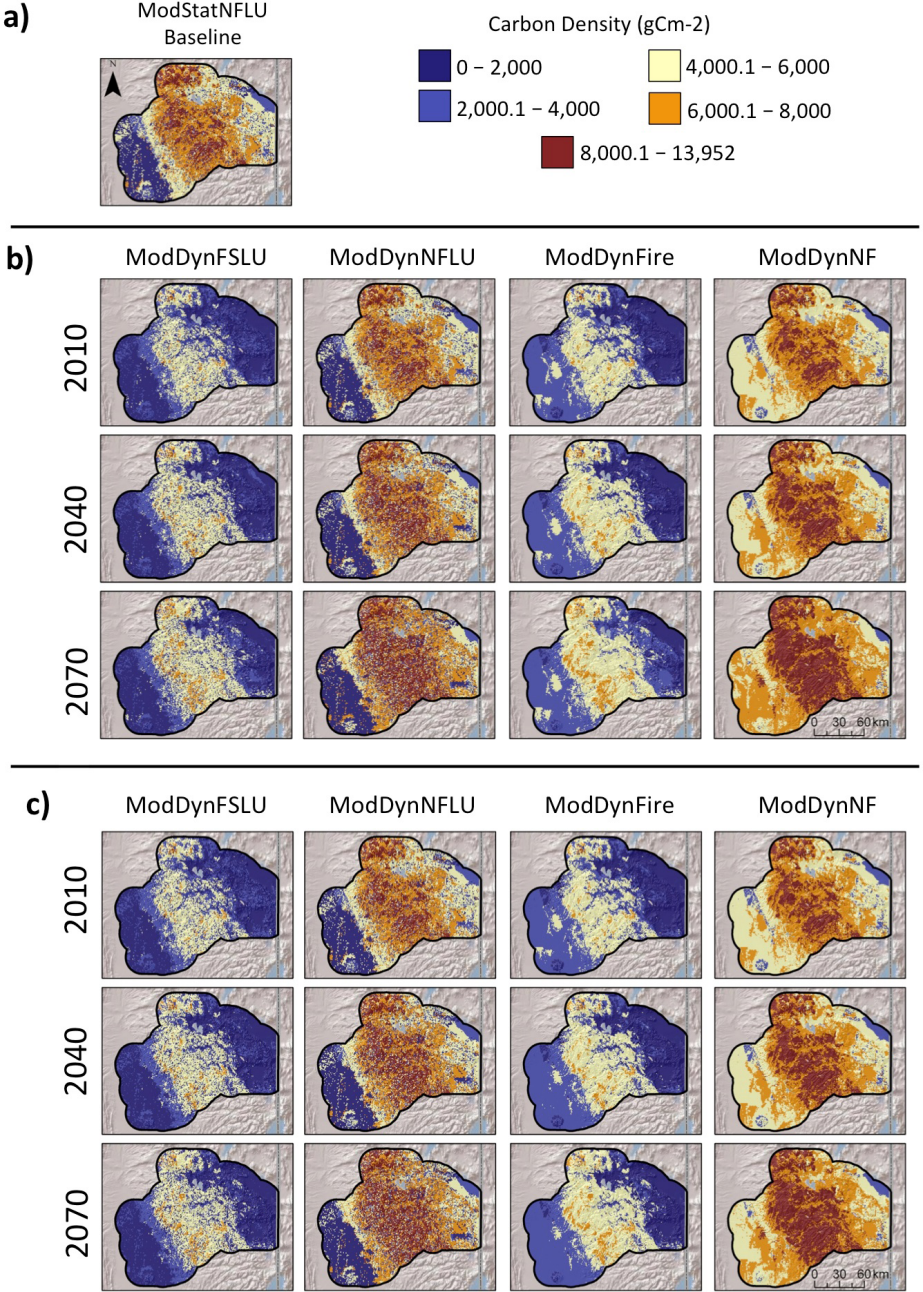
Mean annual carbon in aboveground dead wood from MC2 runs for selected scenarios with the a) CNRM, and b) MIROC climate futures.

**Fig 5:**
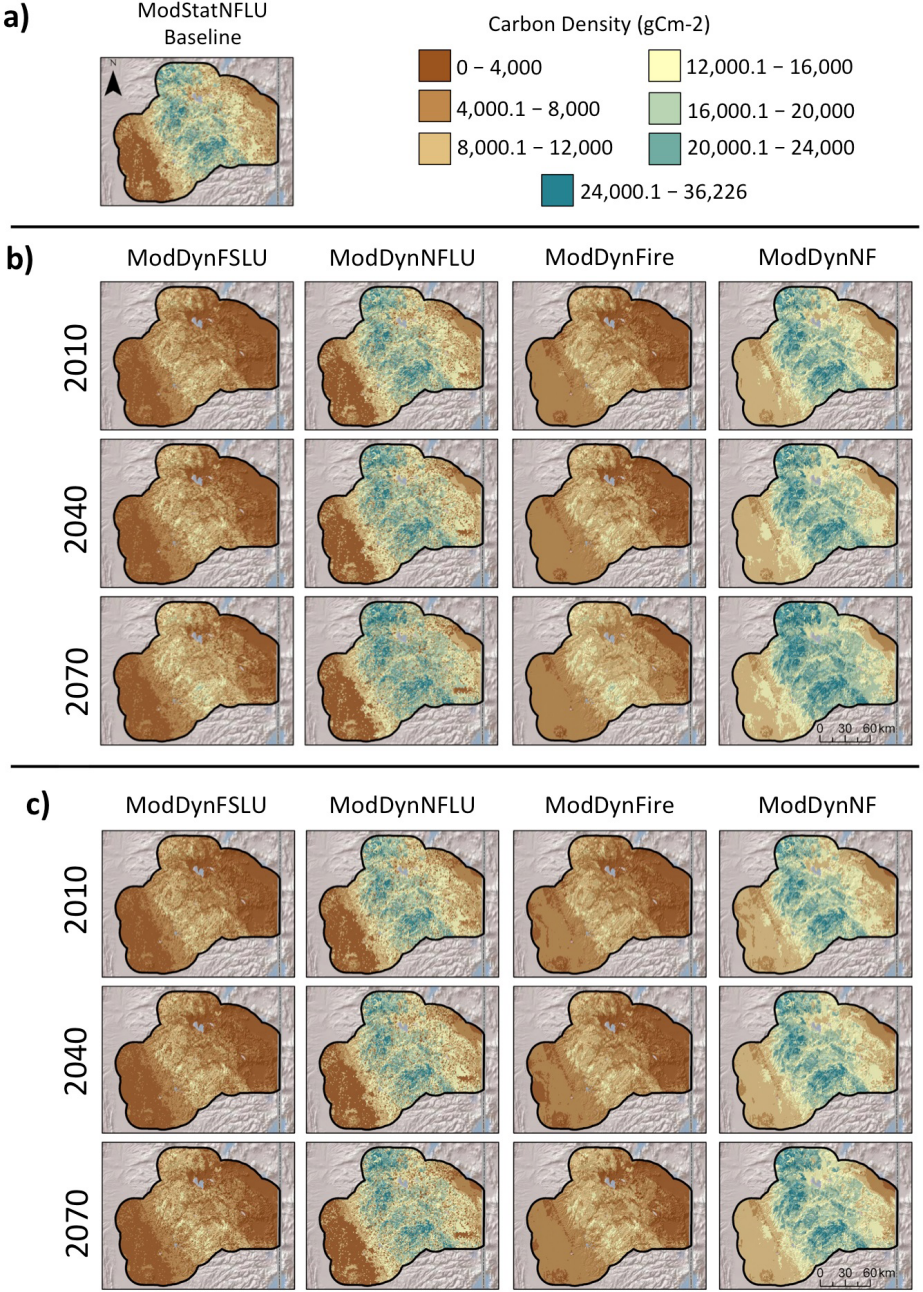
Mean annual carbon in live woody carbon (leaves, boles, branches, and roots) from MC2 runs for selected scenarios with the a) CNRM, and b) MIROC climate futures.

Carbon in above ground dead wood (Fig 4) was much higher in without-fire scenarios than with-fire and was generally higher under CNRM than MIROC. Also, it was much higher at higher elevations. Values for live woody carbon (leaves, boles, branches, and roots) (Fig 5) followed similar patterns as those for aboveground dead wood.

### Baseline fire maps and models

**Fig 6:**
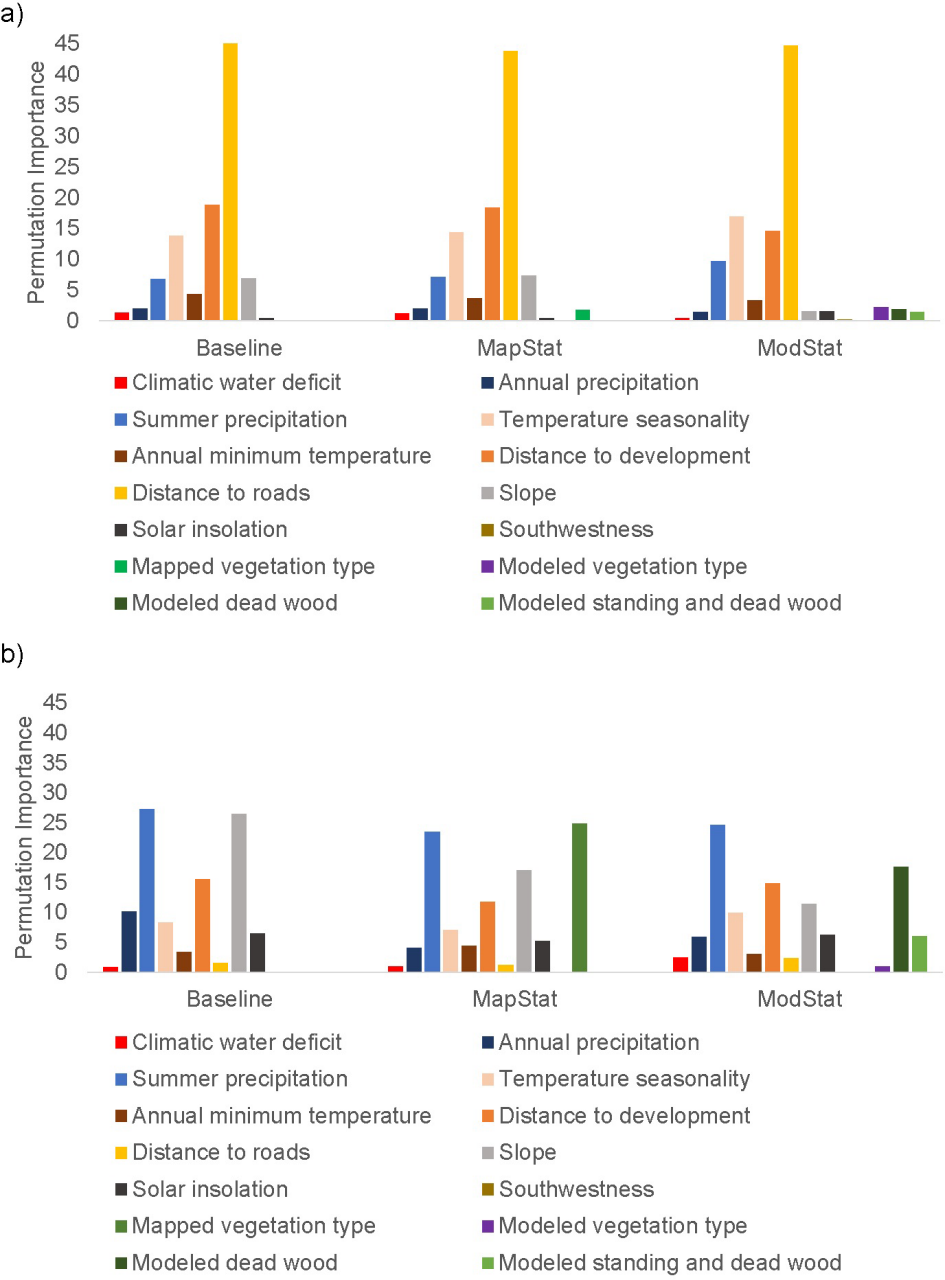
Relative variable importance of explanatory variables for statistical models of a) all fires and b) large fires in the Butte Plumas study area for the three baseline scenarios (1980–2010), including Baseline (no vegetation), MapStat (LANDFIRE existing vegetation type) and ModStat (vegetation type output from dynamic model).

The three baseline scenarios for all fires were similar (Fig 6A). In all three, distance to roads was the most important explanatory variable, with fires more likely to occur in close proximity to roads. The next two top-ranking variables were proximity to development, a negative relationship, and temperature seasonality, with fires more likely to occur with intermediate variation in seasonal temperature. Cooler summer temperatures and lower-percentage slopes were also moderately important. In the two baseline scenarios that included vegetation (MapStat with LANDFIRE and ModStat with modeled vegetation), the vegetation variables were among the lowest-ranking in importance of explanatory variables. The mean cross-validated AUC for all three baseline scenarios was 0.64.

The baseline scenario variable importance for large fires was similar across non-vegetation explanatory variables, although the most important variables for large fires were dissimilar to the ones important for all fires (Fig 6B). The most important non-vegetation variables for large fires included low-moderate summer precipitation, steep slopes, intermediate distance to development, and moderate-to-high climatic water deficit. Temperature variables were much less important than precipitation variables. For the baseline scenarios with vegetation included as explanatory variables, vegetation was either the most important (MapStat) or second-most important (ModStat) variable in the model. If the three vegetation variables are summed in ModStat, then vegetation was the most important variable for that scenario as well. The mean cross-validated AUC for the large fire baselines was 0.75 for NoVeg, 0.79 for MapStat, and 0.76 for ModStat.

### Area and probability of fires under alternative future scenarios

#### All fires

**Fig 7:**
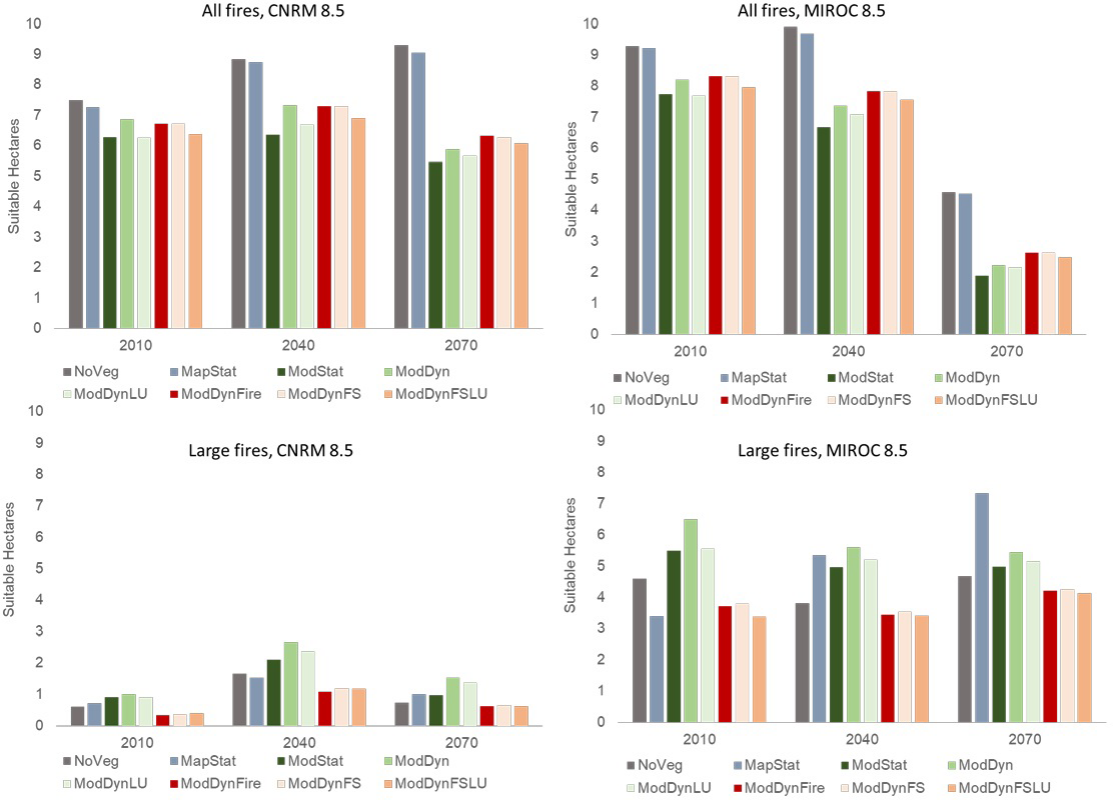
Area predicted suitable for all and large fires for eight vegetation scenarios, three time steps (baseline, mid-century and end-century) and two climate scenarios. Scenario abbreviations are provided in Table 2.

**Fig 8:**
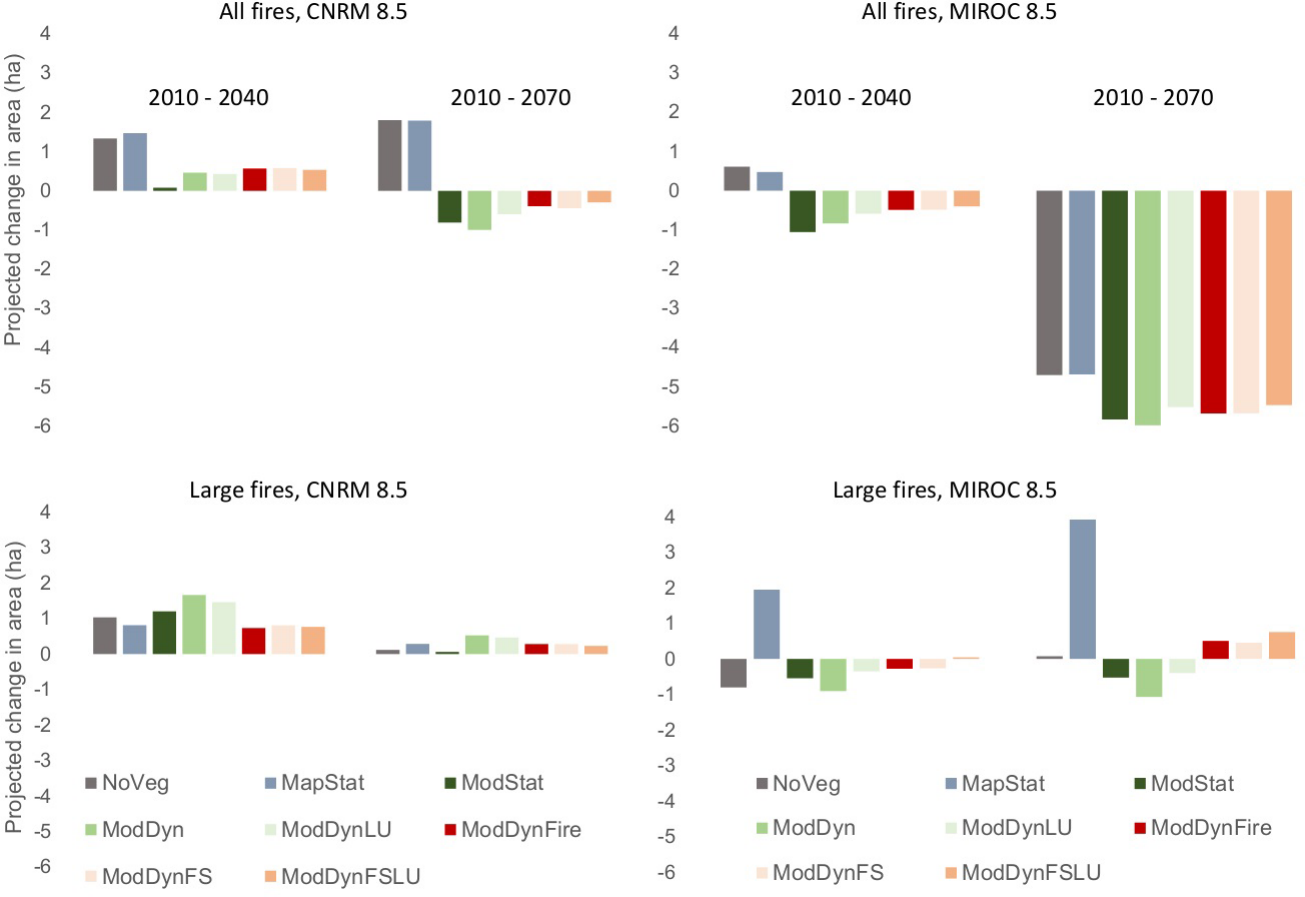
Projected change in suitable area from 2010–2040 and 2010 to 2070 for all and large fires for eight vegetation scenarios and two climate scenarios. Scenario abbreviations are provided in Table 2.

For the all fires projections, there was a clear separation of the NoVeg and MapStat scenarios from the scenarios that incorporated modeled vegetation as input variables. Specifically, there was larger overall predicted area suitable for NoVeg and MapStat for both climate scenarios than there was for scenarios with modeled vegetation (Fig 7). This difference among scenarios was so large that by end-century (2070), there was a projected increase in suitable fire area for NoVeg and MapStat under the CNRM scenario, but the opposite was true using modeled vegetation variables, where fire area was projected to decline (Fig 8). Opposite trends in direction of change were also apparent by the mid-century (2040) for the MIROC scenario. Differences among the modeled vegetation scenarios were subtle, although the scenarios where land use was explicitly modeled resulted in slightly lower predicted area suitable (i.e., in ModDyn vs ModDynLU and in ModDynFS vs ModDynFSLU) (Fig 7).

**Fig 9:**
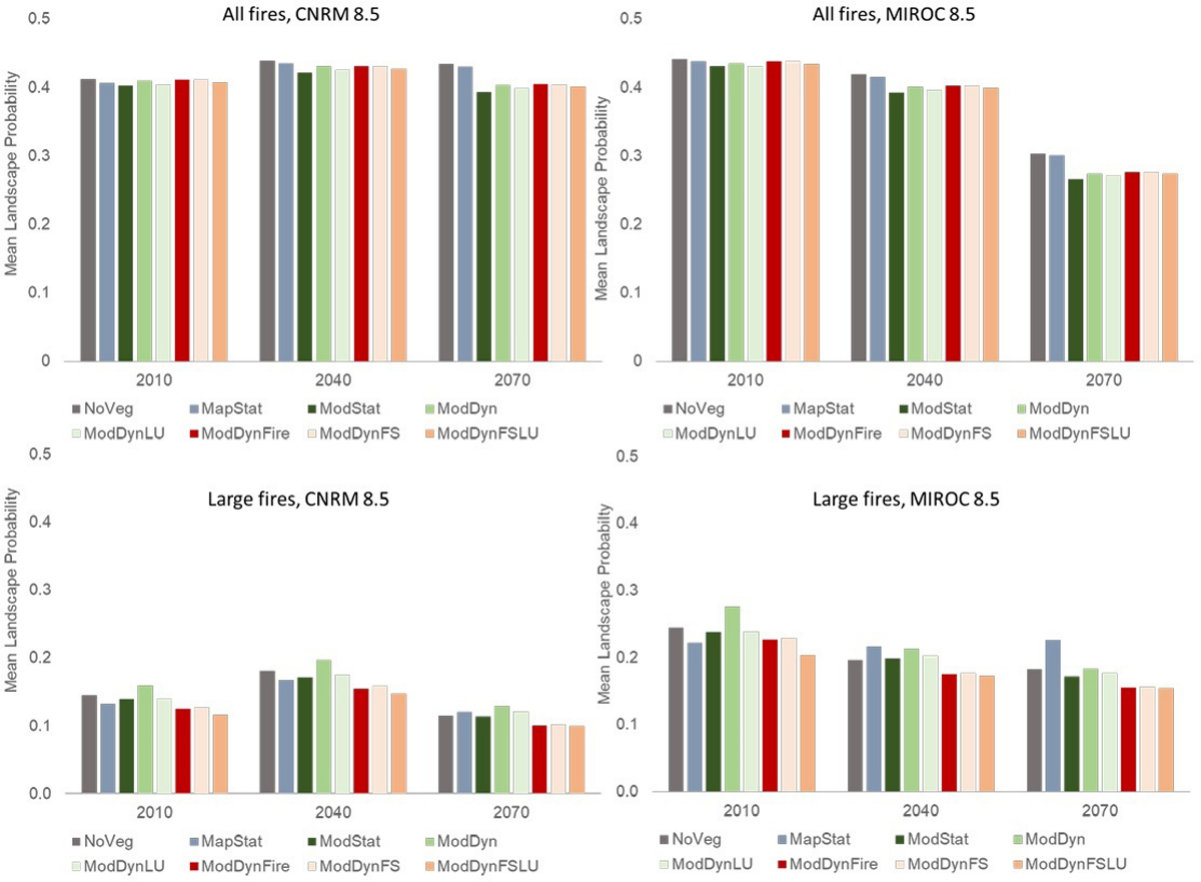
Mean landscape probability of fire for all and large fires for eight vegetation scenarios, three time steps (baseline, mid-century and end-century) and two climate scenarios. Scenario abbreviations are provided in Table 2.

While the projection of larger overall area in the NoVeg and MapStat scenarios was consistent across time periods and climate scenarios, there were also clear differences in overall projected change in fire activity depending on climate scenario. The predicted suitable area for all fires was slightly higher in CNRM than MIROC for baseline and mid-century projections, but the area projected suitable under MIROC for end-century was substantially lower than for CNRM, to the point that there was a projected decline in fire for all scenarios (Figs 7 and 8). The mean landscape-scale probability of large fires was projected to follow a similar trend under the climate scenarios, such that the end-century (2070) MIROC projections not only resulted in lower total suitable area predicted, but the probability of fire in that area was also lower (Fig 9).

#### Large fires

The biggest consistent difference in terms of area predicted suitable for large fires was that the three scenarios that accounted for dynamic wildfire resulted in lower area suitable than all other scenarios (Fig 7). When dynamically modeled vegetation was included without fire or land use change (ModDyn), the predicted suitable area for large fires was the largest among scenarios in all time periods and both climate scenarios, except for end-century (2070) MIROC, where it was second highest. The other two no-fire scenarios using input from the DGVM (ModStat and ModDynLU) also had relatively higher predicted suitable area for large fires than the scenarios without vegetation (NoVeg) or with the static map of existing vegetation (MapStat), except in MIROC mid- and end-century, where MapStat scenario resulted in equal or higher suitable area. As with all fires, the mean probability of fires across the landscape was similar in rank order of.

For the CNRM climate scenario, the projected suitable large fire area increased over time for all vegetation scenarios, mostly in proportion to the total predicted suitable area (Fig 8). There was a larger increase in suitable area projected by mid-century (2040), which slightly diminished by end-century (2070). When projected using the MIROC climate scenario, there were different trends in projections depending upon the vegetation scenario, which did not necessarily follow the rank order of total area predicted suitable. In particular, despite the fact that the dynamic fire scenarios consistently had less total area predicted suitable for large fires, the projected decline over time was lower for these scenarios than all but the MapStat scenario (the only projected to increase) by mid-century. Yet, by end-century, the dynamic fire scenarios (and MapStat) were projected to experience an increased trend of suitable fire area, which was opposite to those without fire.

#### Vegetation versus no vegetation for large fires

**Fig 10:**
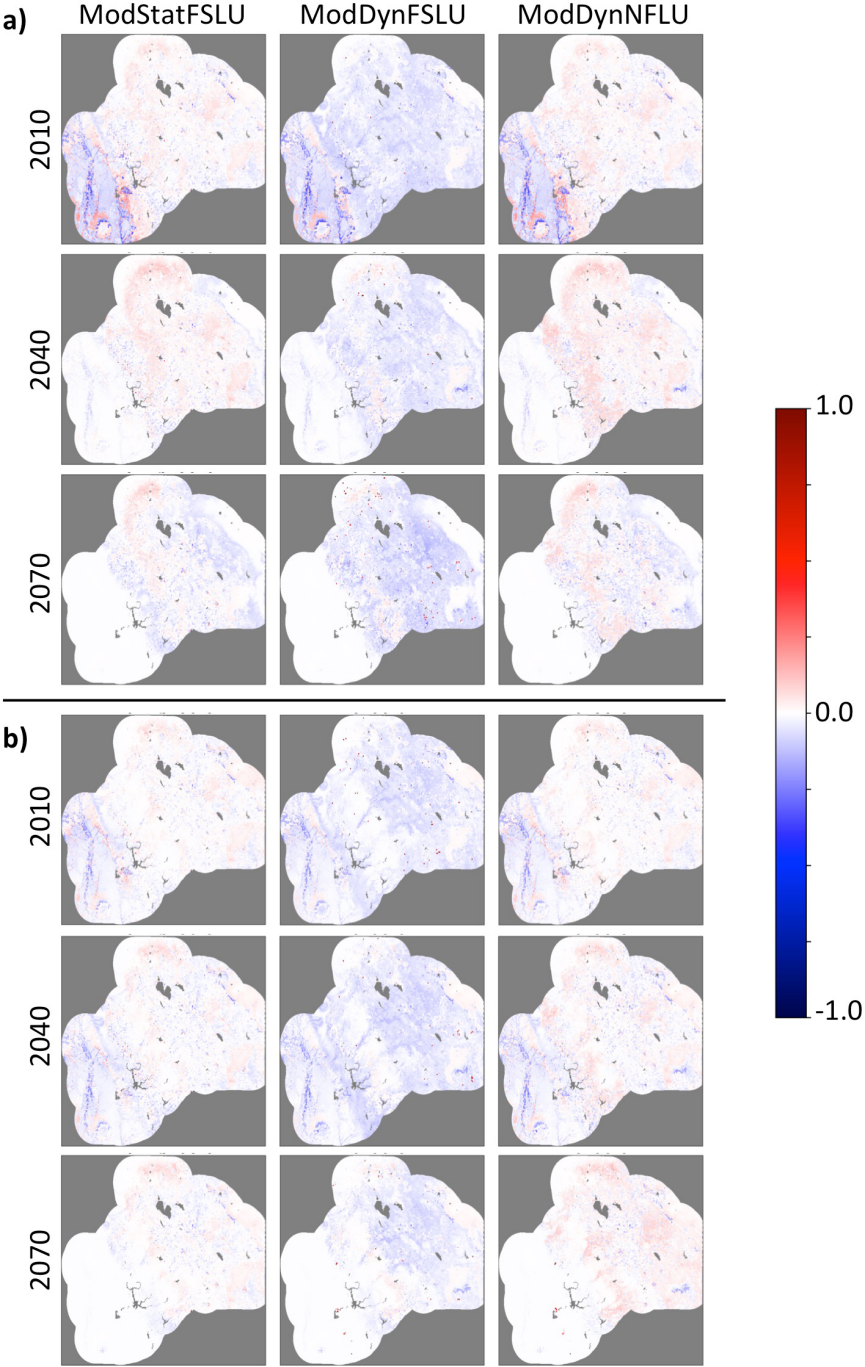
Spatial distribution of large fire probability changes by time period, and scenario for a) CNRM and b) MIROC climate futures. Results were calculated by subtracting NoVeg results from those for each of the vegetation scenarios.

For both the CNRM and MIROC, probability differences in the Central Valley are relatively consistent across scenarios with probabilities generally lower in the early 21^st^ c., lower to unchanged in the mid 21^st^ c., and unchanged in the late 21^st^ c. (Fig 10).

**Fig 11:**
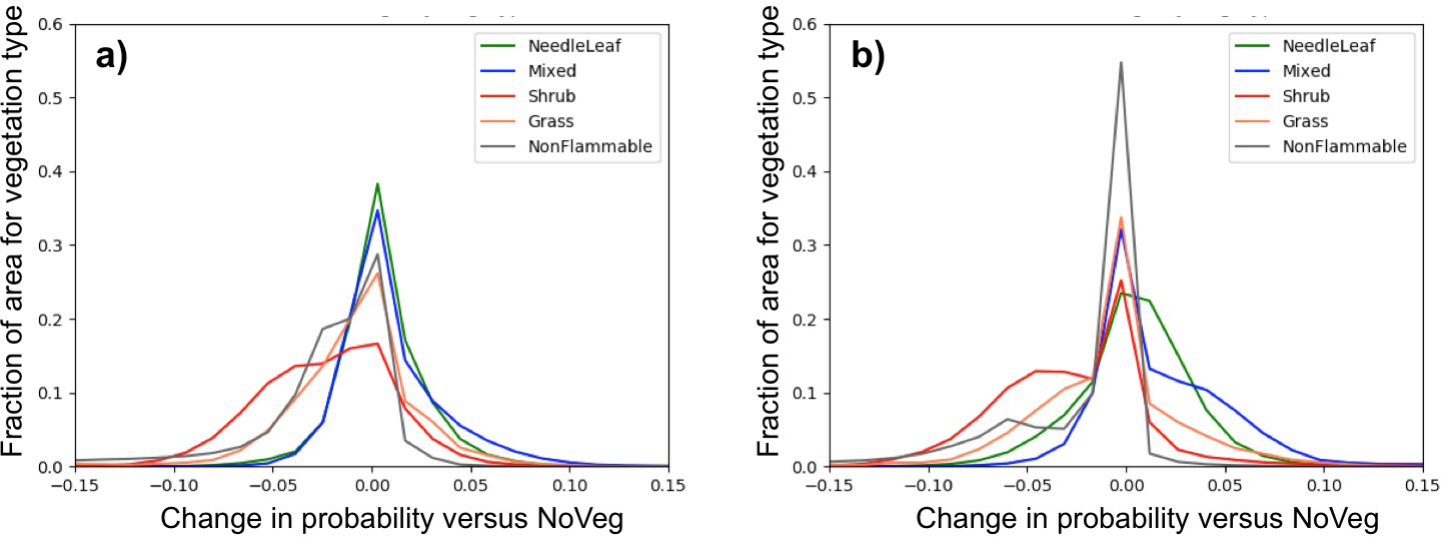
Distribution of large fire probability differences between NoVeg and three scenarios with vegetation (ModStatFSLU, ModDynFSLU, and ModDynNFLU) across all time periods and both climate futures for a) CNRM and b) MIROC climate futures. (Curves are normalized by vegetation type such that the area under a single curve sums to 1.0).

In the Sierra Nevada foothills and mountains, the ModStatLU and ModDynFSLU scenarios fire probability is generally lower under the CNRM climate future and in the early and mid 21^st^ c. under the MIROC climate future (Fig 11). Under the same scenarios, MIROC exhibits a mix of higher and lower probability in the late 21^st^ c. In the same region, fire probability is generally lower for the ModDynFireLU scenario all time periods and both climate futures.

Cumulatively, across time periods, climate future, and vegetation scenario combinations, needleleaf and mixed forest vegetation types were more strongly associated with higher fire probability than lower versus NoVeg, with aggregate mean probability changes of 0.005 and 0.014 respectively (S3 Table and Fig 11). Under several of the combinations, however, each yielded a lower fire probability versus NoVeg. Grass yielded a lower aggregate probability versus NoVeg, -0.009, but yielded a higher probability in 11 of 18 combinations. Shrub and nonflammable yielded the largest lower probabilities versus NoVeg, -0.024 and -0.029 respectively. Each of these was lower than NoVeg in all combinations.

## Discussion

Statistical-correlational models and maps of fire probability are widely used for policy and management decisions, and this study suggests high sensitivity to the treatment of vegetation as a predictor variable, particularly for large fires. Depending on the source of vegetation data and GCM, some models resulted in opposite predictions of directional trends in the extent and probability of future fire. This sensitivity was largely due to the high relative importance of vegetation-related variables in the baseline models and the large amount of vegetation change predicted in the vegetation simulations, particularly those that incorporated wildfire.

Compared to the model without vegetation, the models with the strongest differences in predictions were those that included vegetation dynamics, particularly when there were large changes in nonflammable and shrub vegetation types. For nonflammable vegetation, which primarily increased in the land use scenarios, this is virtually tautological, as the statistical model weighted this strongly towards a low fire probability.

The largest projected declines in large fire probability were associated with increased shrub vegetation, whereas areas with projected increased fire probability were associated with needleleaf and mixed forested areas. Both of these effects are attributable to the effect of fire on vegetation dynamics. The high frequency and intensity of fire that were simulated without fire suppression, and to a lesser extent with fire suppression, were sufficient to replace forests with shrublands throughout much of the Sierra Nevada portion of the study area.

While these projections of vegetation change are substantial, they are also reasonable, as large-scale vegetation type conversion from forest to shrubland with increased high-severity fire is consistently predicted in other studies in the region [65–66]. The lower probability of large fires in models with simulated dynamic fire is also consistent with the well-understood self-limiting effect of wildfire on subsequent fire activity in the Sierra Nevada [67]. Despite the realistic potential for vegetation change to result from increased large-fire activity, the timing of the simulated change here is clearly hypothetical for 2010. That is, because we initiated the simulations with different fire treatments beginning in 1895, the simulated effects on vegetation were already apparent by 2010. However, given that fire suppression effectively minimized large fire activity in the 20^th^ century, the simulation results with no fire better reflect what the current landscape actually looks like.

Another effect of simulated wildfire on resulting large fire probability was via the production of dead wood, which had a high permutation of importance in the baseline model that included it. When wildfire and vegetation dynamics were simulated, dead wood tended to be lower on the landscape, thereby reducing the likelihood of its effect on subsequent fire probability. Conversely, when fire was not explicitly simulated, forests grew and expanded. With this persistence, eventual mortality due to climate stress and senescence produced substantial dead wood that in turn increased future fire probability. The higher change in fire probability associated with mixed forests versus needleleaf forests is attributable to the warmer, drier conditions under which mixed forests grew and thereby affected fuels. Therefore, explicitly accounting for vegetation through a dynamic model affects both fuel abundance and condition.

It has been argued that water balance indices derived from climate variables, particularly climatic water deficit (CWD) and actual evapotranspiration (AET), may indirectly account for the potential changes in vegetation that result from climate change, and can thus serve as vegetation proxies in models predicting future fire probability. These assumptions are rooted in a robust literature relating AET with biomass [68] and CWD with drought, vegetation distributions, and fire [50, 69–70]. These variables have also been significantly related to geographical patterns of fire at broad scales [28,29,71].

We considered both of these variables in our experiment, although AET was highly correlated with other climate variables that had better predictive performance, so we only retained CWD and those variables. Nevertheless, CWD did not end up having substantial variable importance. If CWD and AET sufficiently accounted for vegetation, then models with additional vegetation variables would be expected to provide similar predictions. However, our models with maps of vegetation type and dynamics clearly resulted in different conclusions than the models only using climatic vegetation proxies.

This means that, in our study area, vegetation variables provided additional information that was not accounted for with water balance indices and climate. One of the largest factors that our models accounted for was the effect of dynamic wildfire on fuels, which cannot be predicted by climate variables alone, as other biophysical and anthropogenic factors influence fire and subsequent fuel patterns [17]. Mapped vegetation types may also confer additional information because plant species’ and community distributions, i.e., the realized niche, are determined by more than just climatic effects on physiological response, e.g., they also result from factors such as inter- or intra-specific competition [10]. A final consideration is that prior studies relating CWD and AET to fire patterns have been conducted across larger spatial extents, which may suggest that the effect of these variables is scale-dependent.

Given that the overall performance of our models, reflected in the AUCs, was only moderate to good, there is no way to ascertain which projections of fire are most feasible. There was inherent uncertainty in all of the vegetation variables we considered, including the LANDFIRE map of existing vegetation. While this map may better reflect what is currently on the ground, given that the MC2 vegetation type maps resulted from model simulations beginning in 1895, LANDFIRE has been shown to differ from other maps of existing vegetation [72], and mapped vegetation types in general always connote some level of uncertainty [73].

Assumptions implicit in process-based vegetation models, such as MC2, reflect uncertainties in the processes and drivers of vegetation dynamics. These uncertainties are one of the strongest arguments against the inclusion of vegetation into fire prediction models. However, if, as we have shown, vegetation dynamics influence fire probabilities, these uncertainties are real and their implications should be embraced. While excluding these uncertainties may produce models with greater precision, those models will fail to reflect their own underlying uncertainty. Decisions made based on those models cannot consider the full range of possible futures.

The appropriate scale and complexity of fire modeling within DGVMs is an open question [31]. In addition to showing the value of informing statistical fire probability modeling from vegetation modeling results, this study raises the possibility of integrating statistical fire probability modeling within process-based vegetation models. How to best combine these two approaches is a question with rich potential for exploration. Nevertheless, omitting vegetation dynamics entirely could have large implications for management decisions based on statistical projections of future fire activity.

## Supporting information

S1 Table Classification of LANDFIRE existing and MC2 dynamic vegetation types into broad classes. (DOCX)

S2 Table Values for climate variables used in statistical models for the study area and broken out by elevation. (CWD: climatic water deficit; PPT_ANN: annual precipitation; PPT_SUMM: summer precipitation; TMEAN_COV: temperature seasonality; TMIN: annual minimum temperature; Min: minimum, Max: maximum; StDev: standard deviation).
(DOCX)

S3 Table Change in large fire probability versus the NoVeg scenario by scenario, time period, climate future, and vegetation type. (SD: standard deviation; Num Cells: number of cells within the study area with specified vegetation type).
(DOCX)
