# Supplementary material for: Mapping future fire probability under climate change: Does vegetation matter?
Source: PLoS One. 2018 Aug 6;13(8):e0201680. doi: 10.1371/journal.pone.0201680 (PMC6078303; doi:10.1371/journal.pone.0201680)
Supplement: S1 Table — (DOCX) [file pone.0201680.s001.docx]

|  | S1 Table. Classification of LANDFIRE existing and MC2 dynamic vegetation types into broad classes | |
| --- | --- | --- |
|  | **Initial type** | **Reclassified type** |
| LANDFIRE | Artemisia tridentata ssp. vaseyana Shrubland Alliance | Shrubland |
|  | Barren | Non-flammable |
|  | California Annual Grassland | Grassland |
|  | California Central Valley Mixed Oak Savanna | Mixed and broadleaf |
|  | California Central Valley Riparian Forest and Woodland | Mixed and broadleaf |
|  | California Central Valley Riparian Herbaceous | Grassland |
|  | California Lower Montane Blue Oak Forest and Woodland | Mixed and broadleaf |
|  | California Lower Montane Blue Oak_Foothill Pine Forest and Woodland | Mixed and broadleaf |
|  | California Lower Montane Foothill Pine Woodland and Savanna | Mixed and broadleaf |
|  | California Mesic Chaparral | Shrubland |
|  | California Montane Jeffrey Pine(_Ponderosa Pine) Woodland | Mixed and broadleaf |
|  | California Montane Riparian Systems | Mixed and broadleaf |
|  | California Montane Woodland and Chaparral | Shrubland |
|  | California Xeric Serpentine Chaparral | Shrubland |
|  | Developed_High Intensity | Non-flammable |
|  | Developed_Low Intensity | Non-flammable |
|  | Developed_Medium Intensity | Non-flammable |
|  | Developed_Roads | Non-flammable |
|  | Great Basin Pinyon_Juniper Woodland | Needleleaf |
|  | Great Basin Semi_Desert Chaparral | Shrubland |
|  | Great Basin Xeric Mixed Sagebrush Shrubland | Shrubland |
|  | Inter_Mountain Basins Aspen_Mixed Conifer Forest and Woodland | Mixed and broadleaf |
|  | Inter_Mountain Basins Big Sagebrush Shrubland | Shrubland |
|  | Inter_Mountain Basins Big Sagebrush Steppe | Shrubland |
|  | Inter_Mountain Basins Curl_leaf Mountain Mahogany Woodland | Mixed and broadleaf |
|  | Inter_Mountain Basins Greasewood Flat | Shrubland |
|  | Inter_Mountain Basins Mixed Salt Desert Scrub | Shrubland |
|  | Inter_Mountain Basins Montane Riparian Forest and Woodland | Mixed and broadleaf |
|  | Inter_Mountain Basins Montane Riparian Shrubland | Shrubland |
|  | Inter_Mountain Basins Montane Sagebrush Steppe | Shrubland |
|  | Inter_Mountain Basins Semi_Desert Grassland | Grassland |
|  | Inter_Mountain Basins Semi_Desert Shrub_Steppe | Shrubland |
|  | Inter_Mountain Basins Sparsely Vegetated Systems | Non-flammable |
|  | Inter_Mountain Basins Sparsely Vegetated Systems II | Non-flammable |
|  | Introduced Upland Vegetation_Annual Grassland | Grassland |
|  | Introduced Upland Vegetation_Annual and Biennial Forbland | Grassland |
|  | Introduced Upland Vegetation_Perennial Grassland and Forbland | Grassland |
|  | Mediterranean California Dry_Mesic Mixed Conifer Forest and Woodland | Needleleaf |
|  | Mediterranean California Lower Montane Conifer Forest and Woodland | Needleleaf |
|  | Mediterranean California Mesic Mixed Conifer Forest and Woodland | Needleleaf |
|  | Mediterranean California Mesic Serpentine Woodland and Chaparral | Needleleaf |
|  | Mediterranean California Mixed Evergreen Forest | Needleleaf |
|  | Mediterranean California Mixed Oak Woodland | Mixed and broadleaf |
|  | Mediterranean California Red Fir Forest | Needleleaf |
|  | Mediterranean California Sparsely Vegetated Systems | Shrubland |
|  | Mediterranean California Sparsely Vegetated Systems II | Shrubland |
|  | Mediterranean California Subalpine Meadow | Grassland |
|  | Mediterranean California Subalpine Woodland | Needleleaf |
|  | North Pacific Montane Grassland | Grassland |
|  | Northern California Mesic Subalpine Woodland | Needleleaf |
|  | Northern and Central California Dry_Mesic Chaparral | Shrubland |
|  | Open Water | Non-flammable |
|  | Pacific Coastal Marsh Systems | Non-flammable |
|  | Quarries_Strip Mines_Gravel Pits | Non-flammable |
|  | Quercus garryana Woodland Alliance | Mixed and broadleaf |
|  | Rocky Mountain Aspen Forest and Woodland | Mixed and broadleaf |
|  | Rocky Mountain Subalpine_Montane Mesic Meadow | Grassland |
|  | Sierra Nevada Alpine Dwarf_Shrubland | Shrubland |
|  | Sierra Nevada Subalpine Lodgepole Pine Forest and Woodland | Needleleaf |
|  | Sonora_Mojave Semi_Desert Chaparral | Shrubland |
|  | Western Cool Temperate Close Grown Crop | Non-flammable |
|  | Western Cool Temperate Developed Ruderal Deciduous Forest | Non-flammable |
|  | Western Cool Temperate Developed Ruderal Evergreen Forest | Non-flammable |
|  | Western Cool Temperate Developed Ruderal Grassland | Non-flammable |
|  | Western Cool Temperate Developed Ruderal Shrubland | Non-flammable |
|  | Western Cool Temperate Fallow/Idle Cropland | Non-flammable |
|  | Western Cool Temperate Orchard | Non-flammable |
|  | Western Cool Temperate Pasture and Hayland | Non-flammable |
|  | Western Cool Temperate Row Crop | Non-flammable |
|  | Western Cool Temperate Undeveloped Ruderal Grassland | Grassland |
|  | Western Cool Temperate Undeveloped Ruderal Shrubland | Shrubland |
|  | Western Cool Temperate Urban Deciduous Forest | Non-flammable |
|  | Western Cool Temperate Urban Evergreen Forest | Non-flammable |
|  | Western Cool Temperate Urban Herbaceous | Non-flammable |
|  | Western Cool Temperate Urban Mixed Forest | Non-flammable |
|  | Western Cool Temperate Urban Shrubland | Non-flammable |
|  | Western Cool Temperate Vineyard | Non-flammable |
|  | Western Cool Temperate Wheat | Non-flammable |
|  | Western Warm Temperate Bush fruit and berries | Non-flammable |
|  | Western Warm Temperate Close Grown Crop | Non-flammable |
|  | Western Warm Temperate Developed Ruderal Deciduous Forest | Non-flammable |
|  | Western Warm Temperate Developed Ruderal Evergreen Forest | Non-flammable |
|  | Western Warm Temperate Developed Ruderal Grassland | Non-flammable |
|  | Western Warm Temperate Developed Ruderal Shrubland | Non-flammable |
|  | Western Warm Temperate Fallow/Idle Cropland | Non-flammable |
|  | Western Warm Temperate Orchard | Non-flammable |
|  | Western Warm Temperate Pasture and Hayland | Non-flammable |
|  | Western Warm Temperate Row Crop | Non-flammable |
|  | Western Warm Temperate Row Crop _ Close Grown Crop | Non-flammable |
|  | Western Warm Temperate Undeveloped Ruderal Deciduous Forest | Mixed and broadleaf |
|  | Western Warm Temperate Undeveloped Ruderal Grassland | Grassland |
|  | Western Warm Temperate Undeveloped Ruderal Shrubland | Grassland |
|  | Western Warm Temperate Urban Deciduous Forest | Non-flammable |
|  | Western Warm Temperate Urban Evergreen Forest | Non-flammable |
|  | Western Warm Temperate Urban Herbaceous | Non-flammable |
|  | Western Warm Temperate Urban Mixed Forest | Non-flammable |
|  | Western Warm Temperate Urban Shrubland | Non-flammable |
|  | Western Warm Temperate Vineyard | Non-flammable |
|  | Western Warm Temperate Wheat | Non-flammable |
| MC2 |  |  |
|  | Tundra | Non-flammable |
|  | Subalpine | Needleleaf |
|  | Maritime Evergreen Needleleaf Forest | Needleleaf |
|  | Temperate Evergreen Needleleaf Forest | Needleleaf |
|  | Temperate Cool Mixed Forest | Mixed and broadleaf |
|  | Temperate Warm Mixed Forest | Mixed and broadleaf |
|  | Temperate Evergreen Needleleaf Woodland | Needleleaf |
|  | Temperate Cool Mixed Woodland | Mixed and broadleaf |
|  | Temperate Warm Mixed Woodland | Mixed and broadleaf |
|  | Temperate Shrubland | Shrubland |
|  | Temperate Grassland | Grassland |
|  | Subtropical Mixed Forest | Mixed and broadleaf |
|  | Subtropical Mixed Woodland | Mixed and broadleaf |
|  | Subtropical Shrubland | Shrubland |
|  | Subtropical Grassland | Grassland |
|  | Cool Needleleaf Forest | Needleleaf |
|  | Agriculture Grazing | Non-flammable |
|  | Developed | Non-flammable |
|  | Perennial Agriculture | Non-flammable |
