# Supplementary material for: Mapping future fire probability under climate change: Does vegetation matter?
Source: PLoS One. 2018 Aug 6;13(8):e0201680. doi: 10.1371/journal.pone.0201680 (PMC6078303; doi:10.1371/journal.pone.0201680)
Supplement: S2 Table — (CWD: climatic water deficit; PPT_ANN: annual precipitation; PPT_SUMM: summer precipitation; TMEAN_COV: temperature seasonality; TMIN: annual minimum temperature; Min: minimum, Max: maximum; StDev: standard deviation). (DOCX) [file pone.0201680.s002.docx]

**Table S2.** Values for climate variables used in statistical models for the study area and broken out by elevation. (CWD: climatic water deficit; PPT_ANN: annual precipitation; PPT_SUMM: summer precipitation; TMEAN_COV: temperature seasonality; TMIN: annual minimum temperature; Min: minimum, Max: maximum; StDev: standard deviation)

| Entire Study Area | |  |  |  |  |  |  |  |
| --- | --- | --- | --- | --- | --- | --- | --- | --- |
|  |  |  | CNRM |  |  | MIROC |  |  |
|  |  | BASELINE | 2010 | 2040 | 2070 | 2010 | 2040 | 2070 |
| Mean | CWD | 682.170 | 655.587 | 711.414 | 754.979 | 684.756 | 770.764 | 849.214 |
|  | PPT_ANN | 933.760 | 1228.422 | 1242.427 | 1326.908 | 949.910 | 811.406 | 777.320 |
|  | PPT_SUMM | 18.947 | 32.055 | 29.814 | 36.065 | 30.746 | 37.525 | 31.291 |
|  | TMEAN_COV | 0.024 | 0.023 | 0.024 | 0.023 | 0.024 | 0.025 | 0.026 |
|  | TMIN | -2.474 | -1.398 | -0.690 | 1.662 | -2.257 | -0.548 | 1.256 |
| Min | CWD | 0.000 | 2.170 | 21.040 | 64.745 | 7.110 | 34.870 | 118.734 |
|  | PPT_ANN | 131.430 | 197.363 | 197.623 | 219.545 | 153.537 | 136.733 | 128.745 |
|  | PPT_SUMM | 4.640 | 6.280 | 6.067 | 6.369 | 6.053 | 7.280 | 6.172 |
|  | TMEAN_COV | 0.021 | 0.020 | 0.021 | 0.020 | 0.021 | 0.022 | 0.024 |
|  | TMIN | -12.144 | -10.237 | -9.473 | -6.688 | -11.638 | -9.773 | -8.033 |
| Max | CWD | 1021.770 | 1037.517 | 1054.300 | 1100.621 | 1045.373 | 1110.347 | 1159.455 |
|  | PPT_ANN | 2903.860 | 3666.017 | 3749.333 | 4012.204 | 2852.317 | 2490.387 | 2430.710 |
|  | PPT_SUMM | 57.150 | 108.880 | 91.063 | 119.638 | 95.503 | 111.637 | 104.572 |
|  | TMEAN_COV | 0.028 | 0.027 | 0.027 | 0.027 | 0.028 | 0.029 | 0.030 |
|  | TMIN | 4.545 | 5.317 | 5.849 | 8.071 | 4.581 | 6.232 | 8.067 |
| StDev | CWD | 156.823 | 167.258 | 154.952 | 153.500 | 163.329 | 161.661 | 149.829 |
|  | PPT_ANN | 501.064 | 654.486 | 664.387 | 703.833 | 506.946 | 425.366 | 408.683 |
|  | PPT_SUMM | 7.702 | 14.593 | 13.284 | 17.800 | 13.818 | 16.716 | 14.283 |
|  | TMEAN_COV | 0.001 | 0.001 | 0.001 | 0.001 | 0.001 | 0.001 | 0.001 |
|  | TMIN | 4.216 | 4.126 | 3.988 | 3.771 | 4.258 | 4.198 | 4.211 |
|  |  |  |  |  |  |  |  |  |
| Elevation <= 1000 m | |  |  |  |  |  |  |  |
|  |  |  | CNRM |  |  | MIROC |  |  |
|  |  | BASELINE | 2010 | 2040 | 2070 | 2010 | 2040 | 2070 |
| Mean | CWD | 806.176 | 789.472 | 827.049 | 863.535 | 820.212 | 898.684 | 957.027 |
|  | PPT_ANN | 826.056 | 1070.951 | 1080.711 | 1142.094 | 824.857 | 702.429 | 673.717 |
|  | PPT_SUMM | 11.724 | 17.036 | 16.053 | 17.342 | 16.263 | 20.629 | 17.025 |
|  | TMEAN_COV | 0.023 | 0.022 | 0.023 | 0.023 | 0.023 | 0.024 | 0.025 |
|  | TMIN | 2.328 | 3.301 | 3.835 | 5.899 | 2.555 | 4.214 | 6.062 |
| Min | CWD | 283.730 | 245.193 | 296.493 | 318.255 | 284.123 | 327.967 | 405.869 |
|  | PPT_ANN | 412.820 | 520.390 | 517.390 | 547.679 | 391.847 | 332.920 | 318.597 |
|  | PPT_SUMM | 4.690 | 6.280 | 6.067 | 6.369 | 6.053 | 7.280 | 6.172 |
|  | TMEAN_COV | 0.021 | 0.020 | 0.021 | 0.021 | 0.021 | 0.022 | 0.024 |
|  | TMIN | -4.320 | -3.167 | -2.421 | 0.001 | -4.084 | -2.439 | -0.579 |
| Max | CWD | 1018.500 | 1031.340 | 1050.263 | 1095.066 | 1039.770 | 1105.170 | 1156.238 |
|  | PPT_ANN | 2011.830 | 2553.570 | 2575.000 | 2729.369 | 1956.413 | 1633.640 | 1561.766 |
|  | PPT_SUMM | 25.830 | 40.320 | 37.633 | 44.341 | 38.800 | 49.763 | 41.324 |
|  | TMEAN_COV | 0.025 | 0.024 | 0.025 | 0.024 | 0.025 | 0.026 | 0.028 |
|  | TMIN | 4.545 | 5.317 | 5.849 | 8.071 | 4.581 | 6.232 | 8.067 |
| StDev | CWD | 103.716 | 121.853 | 111.675 | 117.662 | 107.127 | 112.992 | 106.643 |
|  | PPT_ANN | 380.704 | 510.658 | 518.981 | 548.875 | 395.180 | 333.610 | 319.875 |
|  | PPT_SUMM | 4.636 | 7.538 | 7.075 | 7.801 | 7.229 | 9.713 | 7.708 |
|  | TMEAN_COV | 0.001 | 0.000 | 0.000 | 0.001 | 0.000 | 0.000 | 0.000 |
|  | TMIN | 1.136 | 1.133 | 1.070 | 1.009 | 1.122 | 1.137970828 | 1.152 |
|  |  |  |  |  |  |  |  |  |
| Elevation > 1000 m | |  |  |  |  |  |  |  |
|  |  |  | CNRM |  |  | MIROC |  |  |
|  |  | BASELINE | 2010 | 2040 | 2070 | 2010 | 2040 | 2070 |
| Mean | CWD | 602.366 | 572.989 | 638.841 | 684.676 | 601.485 | 691.878 | 781.585 |
|  | PPT_ANN | 1048.191 | 1378.919 | 1401.034 | 1502.863 | 1068.818 | 915.589 | 878.013 |
|  | PPT_SUMM | 23.026 | 40.953 | 38.036 | 47.007 | 39.681 | 48.027 | 40.534 |
|  | TMEAN_COV | 0.024 | 0.023 | 0.024 | 0.023 | 0.024 | 0.025 | 0.027 |
|  | TMIN | -5.296 | -4.181 | -3.383 | -0.858 | -5.109 | -3.393 | -1.610 |
| Min | CWD | 0.000 | 2.170 | 21.040 | 64.745 | 7.110 | 34.870 | 118.734 |
|  | PPT_ANN | 133.760 | 198.677 | 199.367 | 221.041 | 154.587 | 137.770 | 129.828 |
|  | PPT_SUMM | 10.830 | 19.560 | 18.040 | 23.138 | 19.353 | 21.437 | 17.793 |
|  | TMEAN_COV | 0.021 | 0.020 | 0.021 | 0.020 | 0.021 | 0.022 | 0.024 |
|  | TMIN | -12.144 | -10.224 | -9.330 | -6.577 | -10.870 | -9.166 | -7.667 |
| Max | CWD | 943.660 | 940.447 | 993.657 | 1044.079 | 946.383 | 1041.963 | 1123.638 |
|  | PPT_ANN | 2903.860 | 3666.017 | 3749.333 | 4012.204 | 2852.317 | 2490.387 | 2430.710 |
|  | PPT_SUMM | 57.150 | 108.880 | 91.063 | 113.579 | 95.503 | 111.637 | 104.572 |
|  | TMEAN_COV | 0.028 | 0.027 | 0.027 | 0.027 | 0.028 | 0.029 | 0.030 |
|  | TMIN | 1.390 | 2.501 | 3.091 | 5.496 | 1.685 | 3.244 | 4.963 |
| StDev | CWD | 127.978 | 128.892 | 128.075 | 125.299 | 129.211 | 136.494 | 132.734 |
|  | PPT_ANN | 559.909 | 709.446 | 725.151 | 760.877 | 551.448 | 466.877 | 450.487 |
|  | PPT_SUMM | 6.529 | 11.317 | 9.822 | 12.260 | 10.483 | 12.363 | 11.809 |
|  | TMEAN_COV | 0.001 | 0.001 | 0.001 | 0.001 | 0.001 | 0.001 | 0.001 |
|  | TMIN | 2.275 | 2.169 | 2.145 | 2.061 | 2.272 | 2.203 | 2.157 |
