# Supplementary material for: Mapping future fire probability under climate change: Does vegetation matter?
Source: PLoS One. 2018 Aug 6;13(8):e0201680. doi: 10.1371/journal.pone.0201680 (PMC6078303; doi:10.1371/journal.pone.0201680)
Supplement: S3 Table — (SD: standard deviation; Num Cells: number of cells within the study area with specified vegetation type). (DOCX) [file pone.0201680.s003.docx]

**S3 Table.** Change in large fire probability versus the NoVeg scenario by scenario, time period, climate future, and vegetation type. (SD: standard deviation; Num Cells: number of cells within the study area with specified vegetation type).

| **CNRM** | | | | | | | | | | | | | | | |
| --- | --- | --- | --- | --- | --- | --- | --- | --- | --- | --- | --- | --- | --- | --- | --- |
|  | Needleleaf | |  | Mixed |  |  | Shrubland | |  | Grassland | |  | Nonflammable | |  |
|  | Mean | SD | Num Cells | Mean | SD | Num Cells | Mean | SD | Num Cells | Mean | SD | Num Cells | Mean | SD | Num Cells |
| ModStatNFLU 2010 | 0.004 | 0.018 | 159385 | 0.014 | 0.033 | 24216 | -0.007 | 0.038 | 68035 | 0.011 | 0.053 | 2405 | -0.038 | 0.049 | 53946 |
| ModStatNFLU 2040 | 0.002 | 0.021 | 159385 | 0.004 | 0.034 | 24216 | -0.016 | 0.040 | 68035 | 0.011 | 0.055 | 2405 | -0.038 | 0.048 | 53946 |
| ModStatNFLU 2070 | 0.003 | 0.020 | 159385 | -0.004 | 0.005 | 24216 | -0.008 | 0.028 | 68035 | 0.011 | 0.026 | 2405 | -0.004 | 0.021 | 53946 |
| ModDynFSLU 2010 | -0.008 | 0.014 | 16810 | -0.002 | 0.011 | 10139 | -0.029 | 0.043 | 222595 | -0.015 | 0.106 | 3698 | -0.040 | 0.049 | 54745 |
| ModDynFSLU 2040 | -0.010 | 0.017 | 19680 | 0.005 | 0.015 | 12827 | -0.036 | 0.042 | 217038 | -0.023 | 0.057 | 2603 | -0.041 | 0.050 | 55839 |
| ModDynFSLU 2070 | -0.004 | 0.014 | 26598 | 0.005 | 0.015 | 31992 | -0.023 | 0.035 | 190647 | -0.008 | 0.016 | 1846 | -0.005 | 0.040 | 56904 |
| ModDynNFLU 2010 | 0.005 | 0.018 | 143875 | 0.015 | 0.030 | 39042 | -0.009 | 0.035 | 67916 | -0.017 | 0.034 | 2437 | -0.039 | 0.050 | 54717 |
| ModDynNFLU 2040 | 0.006 | 0.023 | 141158 | 0.018 | 0.034 | 48517 | -0.019 | 0.039 | 60709 | -0.020 | 0.034 | 1780 | -0.041 | 0.051 | 55823 |
| ModDynNFLU 2070 | 0.017 | 0.027 | 116203 | 0.010 | 0.024 | 79351 | -0.008 | 0.029 | 54111 | 0.000 | 0.015 | 1471 | -0.005 | 0.039 | 56851 |
| **CNRM weighted mean** | 0.003 |  |  | 0.006 |  |  | -0.012 |  |  | -0.003 |  |  | -0.017 |  |  |
| **MIROC** |  |  |  |  |  |  |  |  |  |  |  |  |  |  |  |
| ModStatNFLU 2010 | 0.007 | 0.023 | 159385 | 0.048 | 0.057 | 24216 | -0.003 | 0.055 | 68035 | 0.017 | 0.069 | 2405 | -0.075 | 0.077 | 53946 |
| ModStatNFLU 2040 | 0.015 | 0.026 | 159385 | 0.009 | 0.024 | 24216 | -0.014 | 0.032 | 68035 | 0.024 | 0.054 | 2405 | -0.012 | 0.023 | 53946 |
| ModStatNFLU 2070 | -0.008 | 0.030 | 159385 | -0.005 | 0.008 | 24216 | -0.021 | 0.035 | 68035 | 0.004 | 0.035 | 2405 | -0.002 | 0.017 | 53946 |
| ModDynFSLU 2010 | -0.022 | 0.022 | 27955 | -0.001 | 0.020 | 4786 | -0.036 | 0.041 | 216549 | -0.044 | 0.060 | 3929 | -0.077 | 0.075 | 54768 |
| ModDynFSLU 2040 | -0.004 | 0.023 | 11768 | 0.017 | 0.020 | 11444 | -0.030 | 0.033 | 225317 | -0.016 | 0.020 | 3655 | -0.014 | 0.028 | 55803 |
| ModDynFSLU 2070 | -0.012 | 0.027 | 14770 | 0.006 | 0.025 | 14642 | -0.037 | 0.052 | 219076 | -0.015 | 0.052 | 2592 | -0.004 | 0.024 | 56907 |
| ModDynNFLU 2010 | 0.008 | 0.023 | 150507 | 0.045 | 0.052 | 28863 | -0.003 | 0.053 | 70693 | -0.029 | 0.070 | 3219 | -0.075 | 0.077 | 54705 |
| ModDynNFLU 2040 | 0.018 | 0.027 | 132162 | 0.027 | 0.033 | 45889 | -0.014 | 0.033 | 71816 | -0.002 | 0.019 | 2334 | -0.014 | 0.028 | 55786 |
| ModDynNFLU 2070 | -0.004 | 0.031 | 122578 | 0.009 | 0.029 | 58151 | -0.022 | 0.036 | 69076 | -0.003 | 0.016 | 1431 | -0.004 | 0.024 | 56751 |
| **MIROC weighted mean** | 0.010 |  |  | 0.028 |  |  | -0.032 |  |  | -0.016 |  |  | -0.045 |  |  |
| **All scenarios weighted mean** | 0.006 |  |  | 0.017 |  |  | -0.022 |  |  | -0.009 |  |  | -0.031 |  |  |
